# The effect of COVID-19 pandemic in a large series of patients with Takayasu arteritis

**DOI:** 10.55730/1300-0144.5347

**Published:** 2022-02-05

**Authors:** Ali KARAKAŞ, Tuba YÜCE İNEL, Fatoş ÖNEN, İsmail SARI

**Affiliations:** Division of Rheumatology, Department of Internal Medicine, Faculty of Medicine, Dokuz Eylül University, İzmir, Turkey

**Keywords:** Takayasu arteritis, COVID-19, antirheumatic agents, vaccination

## Abstract

**Background/aim:**

Patients with inflammatory rheumatic diseases faced several challenges during the COVID-19 pandemic. Uncertainties such as the lack of evidence regarding the use of immunosuppressive (IS) therapies and deferred patient care because of limited health resources affected negatively on many aspects of treatment decisions and routine follow-up of the patients. In this study, we aimed to investigate the prevalence and severity of SARS-CoV-2 infection, the impact of the pandemic on delays in routine clinical follow-up, changes in IS treatment, and COVID-19 vaccination status of patients with Takayasu arteritis (TAK).

**Materials and methods:**

The study was performed between July and September 2021. TAK patients who registered in our database were investigated with regards to the COVID-19 infection and vaccination status, delays in routine clinical visits, changes in their IS treatments, and flares during the pandemic. Physical examination, laboratory tests, and imaging of the patients were performed and ITAS2010 scores were calculated.

**Results:**

There were 56 adult TAK patients (87.5% female and median age 47 years). A total of 44 (78.6%) patients experienced a delay with routine follow-up visits to their physicians and about 20% of patients stopped their antirheumatic treatments without consulting their physicians. Compared to the pre-COVID-19 pandemic, 16 (28.5%) patients flared. In total group, 13 (23.2%) patients had a mild COVID-19 infection and about 90% of the patients had received the COVID-19 vaccine.

**Conclusion:**

Deferred patient care and disease flares are the most significant problems in TAK patients during the pandemic. The risk of TAK flares may outweigh the risk of COVID-19 infection.

## 1. Introduction

Coronavirus disease 2019 (COVID-19) is a significant health problem with increased mortality and morbidity, particularly for patients with underlying chronic disease [1]. In this respect, patients with inflammatory rheumatic diseases are considered at risk for a severe COVID-19 because of the abnormal immune response and the potential use of the immunosuppressive (IS) therapies [2,3]. Takayasu arteritis (TAK) is a rare form of large-vessel vasculitis mainly affecting young women of Asian ethnicity. Vasculitis causing narrowing, occlusion, or dilation of arteries may affect several organ systems and may lead to numerous symptoms. Systemic IS therapy is required to control disease activity and to prevent complications [4]. The prevalence and impact of COVID-19 on patients with TAK are currently limited [5,6]. Herein, due to the limited number of reports on COVID-19 and TAK, we studied the prevalence and severity of SARS-CoV-2 infection, the impact of the pandemic on delays in routine clinical follow-up, changes in IS treatment, and COVID-19 vaccination status of patients with Takayasu arteritis (TAK).

## 2. Materials and methods

TAK patients classifying the American College of Rheumatology 1990 Takayasu arteritis were included in the study [7]. Patients who provided informed consent were asked the following questions during their routine visits: (1) Have you been diagnosed with COVID-19? (2) Have you regularly been taking your TAK medications? (3) Have you experienced a relapse of your disease? (4) Did you have a delay with routine follow-up visits to your physician? (5) If the answer yes for question number five, how long was it delayed? (6) Have you been vaccinated for COVID-19? and (7) Have you experienced any side effects related to the COVID-19 vaccine? Following the inquiry, the patients were examined by a rheumatologist and disease activity scores ITAS 2010 [8] and Kerr [9] were calculated.

Demographic data, disease characteristics, duration of diagnosis, comorbidities and treatments were recorded. Severe COVID-19 infection was defined if the patient had one of the following: hospitalization with a diagnosis of acute respiratory failure, need for noninvasive ventilation, admission to an intensive care unit, or death [10]. The study was performed between July and September 2021. Ethical approval was obtained from the ethics committee of Dokuz Eylül University School of Medicine and the Ministry of Health, General Directorate of Health Services.

### 2.1. Statistical analysis

Normality was tested by using the Kolmogorov–Smirnov test. Continuous variables were presented as median with interquartile range (IQR). The chi-square test or Fisher’s exact test was used to compare for categorical variables and the Mann–Whitney U test was used for the comparison of the continuous data. For all statistical analyses, a double tailed p-value <0.05 was considered statistically significant. Statistical analysis was carried out by using SPSS v.24.

## 3. Results

Out of 69 adult TAK patients who were registered in our database, 56 (81.2%) were agreed to participate in the study. Five patients died before the pandemic, 8 patients refused to participate in the study (there was no mortality in this group and the clinic data of these patients were not shared). The median (Q1–Q3) age of the study patients was 47 (37–60) years, 49 (87.5%) were female and the median (Q1–Q3) disease duration was nine (4–17) years. The general characteristics of the patients were given in Table 1. There were 44 (78.6%) patients who experienced a delay with their routine follow-up visits and about 20% of the patients stopped their treatments without consulting their physicians. The vast majority of the patients (n = 32, 73%) delayed their visits as they had difficulty getting an appointment during the pandemic. The remainder (n = 12) of the patients did not attend their visits as a concern for contacting the COVID-19 patients. Five (45.5%) out of 11 patients experienced flare after three (2–6) months of stopping their treatments. Compared to the pre-COVID-19 pandemic (before 11.03.2020 when the first COVID-19 case was seen in Turkey), 16 (28.5%) patients flared according to Kerr criteria [9]. Fifty (91%) out of 56 patients were vaccinated for COVID-19. Patients who did not get vaccinated mostly did not have their vaccines as of concern with regards to the side effects of the vaccination (n = 5). Only one patient rejected the vaccine on religious grounds. The detailed data about the disease and COVID-19 vaccination status were summarized in Table 2. Pain at the injection site, fatigue, and headache were the most common side effects of the vaccination. There were no serious adverse events related to the vaccination. A total of 13 (23.2%) patients had a documented COVID-19 infection. All but one patient had both positive PCR tests for COVID-19 and clinical symptoms. Although PCR was negative in one patient, the diagnosis was made with a history of close contact with a COVID-19 patient and clinical symptoms. Eight of them had an infection before the vaccination program started. Three of the patients had an infection after a double dose of Sinovac-CoronaVac COVID-19 vaccine, one after a single dose of Sinovac-CoronaVac COVID-19 vaccine, and the other after a double dose of Pfizer-BioNTech COVID-19 vaccine. Pneumonia was reported in five patients, and two of them required hospitalization. One patient required intensive care support. Three patients had a loss of taste and smell. All patients were given Favipiravir and anticoagulant based on local treatment guidelines^1^ for COVID-19, and their DMARDs were stopped temporarily until two weeks of symptom-free observation. None of the patients required anticytokine therapy but one patient received oral steroids for COVID-19 infection. All patients recovered completely. There was also no mortality in the group of patients who did not participate in the study. Comparison of patients who had COVID-19 vs. without revealed that patients infected with COVID-19 were younger and had fewer comorbid diseases. The comparison of patients with and without COVID-19 was given in Table 3.

## 4. Discussion

In this study, we revealed the following: about one fifth of the TAK patients were infected with the COVID-19, the course of infection was mild in about 95% of the patients and there was no mortality because of COVID-19. The majority of the TAK patients (about 90%) were agreed to vaccinate against COVID-19. During the pandemic about one third of the TAK patients flared and the majority of these were observed in patients whose routine follow-ups were delayed as healthcare resources largely utilized for pandemic control.

IS treatments form the mainstay in controlling disease activity in inflammatory rheumatic diseases (IRDs). However, these medications are also of particular concern for susceptibility to infections. Currently the evidence regarding the use of IS therapies and their impact of COVID-19 infection is limited. An Italian study reported that 2 (3%) out of 67 TAK patients had a confirmed diagnosis of COVID-19 and these patients did not require hospitalization. Therefore authors speculated that the use of IS therapy did not adversely affect the course of COVID-19 [6]. In contrast, another study in patients with large vessel vasculitis (LVV) documented a higher hospitalization and mortality rates suggesting a severe outcome in COVID-19 disease. They reported the incidence of COVID-19 amongst the TAK cohort was 6.3%, and the mortality rate was 1.7% [11]. In another study, among the vasculitis, TAK reported to be the group with the lowest incidence of COVID-19 infection (2.4%, n: 16/662) [12]. In our cohort, the rate of COVID-19 was 23.2% and only about 5% had severe COVID-19 without any mortality. The higher frequency in our series could be explained by the longer pandemic duration compared to other studies. Current evidence suggested that the use of disease modifying antirheumatic drugs (DMARDs) and lower doses of prednisone (<10 mg/daily) did not increase the severity of COVID-19 infection [3,13–15]. However, higher doses of corticosteroids (CS) have been associated with delayed viral clearance from the blood and respiratory tract [16,17]. In our cohort 58.9 % of patients were on CS and but only three patients were taking steroids over 10 mg/day.

Taken together with the low CS doses, younger age and low comorbidities among COVID-19 patients may contributed to the milder outcome for COVID-19 infection in our patients.

In many countries pandemic affected the health system profoundly. Available healthcare resources including outpatient care used to treat and control the COVID-19 infection. This affected the routine follow-ups of the patients who have chronic diseases. In our cohort, nearly 80% of our patients reported that they experienced a delay with their follow-up visits to their physicians. Both the lack of communication between patients and physicians and concern for COVID-19 infection in some patients about 20% of the cohort stopped their medications. This was resulted a disease flare in 45.5% of the patients who discontinued their treatments. Similar to our study, nearly 70% of the vasculitis patients reported delays in their follow-ups and about 10% needed to stop IS therapies [12].

Vaccination is the most effective form of protection against COVID-19. In our country both inactive and mRNA vaccines are present and about 60% of the population has been vaccinated against COVID-19. The vaccination rate among our TAK cohort is 91% and only five patients refused the vaccine for various reasons. There were no mortality in the total TAK cohort as because of COVID-19. It is of concern that vaccination may have an effect on disease flares. Based on self-reports, 5% of patients with autoimmune disease reported deterioration of their disease within two months of vaccination [18]. In our study, we evaluated our patients based on ITAS10 score and there was no deterioration of TAK activity after the vaccination.

The main limitation of the current study was the small sample size and therefore limited statistical power. We also acknowledge that our population was derived from a single center which may also be considered to be a limitation. However, when considering the rarity of the TAK, current data is providing important information to the researchers as there was a substantial number of patients who were being followed up in one of the largest TAK cohorts in the country.

In summary, COVID-19 disease in TAK patients were in mild severity and IS therapy seem not affecting the COVID-19 course. A substantial number of patients who stopped their medications flared and its long-term consequences need to be assessed by large-scale studies. New approaches are required to maximize healthcare access for patients who have chronic diseases during pandemic.

## Figures and Tables

**Table 1:** Clinical and demographic features of patients with Takayasu arteritis.

Demographic features	n = 56
**Age, years, (median, Q1–Q3)**	47 (37–60)
**Female, n (%)**	49 (87.5)
**Disease duration, years (median, Q1–Q3)**	9 (4–17)
**Comorbid disease, n (%)**	27 (48.2)
**Hypertension**	22 (39.3)
**Coronary artery disease**	6 (10.7)
**Pulmonary arterial hypertension**	2 (3.6)
**Cerebrovascular accident**	3 (5.4)
**Diabetes mellitus**	2 (3.6)
**Chronic kidney disease**	3 (5.4)
**Other diseases**	7 (12.5)
**Current and ex-smoker**	19 (37.3)
**Artery involvement type[4] n(%)**	
**Type 1**	16 (28.6)
**Type 2a**	6 (10.7)
**Type 2b**	5 (8.9)
**Type 3**	2 (3.6)
**Type 4**	2 (3.6)
**Type 5**	25 (44.6)
**CRP (mg/L) at last visit, (median, IQR, Q1–Q3)**	4.5 (1.8–9.6)
**ESR (mm/h) at last visit, (median, IQR, Q1–Q3)**	19 (7–29)
**Current treatments n (%)**	
**Prednisolone n (%), median, Q1–Q3 dose (mg)**	33 (58.9), 3 (0–4) mg
**Methotrexate**	19 (33.9)
**Leflunomide**	5 (8.9)
**Mycophenolate mofetil**	10 (17.9)
**Azathioprine**	13 (23.2)
**Cyclophosphamide**	1 (1.8)
**Tocilizumab**	5 (8.9)
**Infliximab**	6 (10.7)
**cs-/bDMARD combination**	10 (17.8)

Continuous data is presented with median (interquartile range, Q1–Q3) values and categorical data are presented as counts (n) and percentages (%), csDMARD, conventional synthetic disease-modifying antirheumatic drug; bDMARD, biological disease-modifying antirheumatic drug; CRP; C-reactive protein; ESR; erythrocyte sedimentation rate.

**Table 2:** Effects of COVID-19 pandemic on patients with Takayasu arteritis.

Patients with a delay regarding to the routine follow-up visits, n (%)	44 (78.6)
Average delay in routine follow-up visits since the beginning of COVID-19 pandemic (months), median, Q1–Q3	3.5 (2.25–9)
Patients who do not receive their treatment regularly due to COVID-19 concern	11 (19.6)
Disease relapse compared to the prepandemic time, n (%)	16 (28.6)
Progression in acute phase reactants, n (%)	14 (25)
Progression in vascular involvement (according to MR angiography or Doppler ultrasound findings) n (%)	15 (26.8)
Patients diagnosed with COVID-19 disease, n (%), prevaccination / postvaccination	13 (23.2), 8/5
Vaccination status, n (%)	50 (90.9)
Sinovac-CoronaVac COVID-19 vaccine single dose	2 (4)
Sinovac-CoronaVac COVID-19 vaccine double dose	9 (18)
Sinovac-CoronaVac COVID-19 vaccine triple dose	6 (12)
Pfizer-BioNTech COVID-19 Vaccine single dose	1 (2)
Pfizer-BioNTech COVID-19 Vaccine double dose	14 (28)
Sinovac-CoronaVac COVID-19 vaccine double dose + Pfizer-BioNTech COVID-19 Vaccine single dose	17 (34)
Sinovac-CoronaVac COVID-19 vaccine double dose + Pfizer-BioNTech COVID-19 Vaccine double dose	1 (2)

Continuous data is presented with median (interquartile range, Q1–Q3) values and categorical data are presented as counts (n) and percentages (%).

**Table 3:** Comparison of patients diagnosed with and without COVID-19 disease.

Variables	COVID-19 (+) patients n = 13	COVID-19 (−) patients n = 43	p value
**Age, mean ± SD**	39.7 ± 10.5	48.7 ± 14.4	**0.042**
**Female, n (%)**	11 (84.6)	38 (88.4)	0.720
**Disease duration, years, median (IQR, Q1–Q3)**	11 (5–12)	7 (3–18)	0.892
**Disease duration, years, Mean ± SD**	11.1 ± 9.5	9.3 ± 4.5	0.369
**CRP (mg / L) at last visit, (median, IQR, Q1–Q3)**	1.4 (0.8–7)	3.5 (1.1–7.6)	0.285
**ESR (mm/h) at last visit, (median, IQR, Q1–Q3)**	11 (4–19.5)	14.5 (7–25.3)	0.312
**ITAS2010 score at last visit**	2 (0–6)	3 (0–5)	0.911
**Current treatments**			
**Prednisolone**	8 (61.5)	25 (58.1)	0.827
**Methotrexate**	2 (15.4)	17 (39.5)	0.107
**Leflunomide**	0 (0)	5 (11.6)	0.198
**Mycophenolate mofetil**	4 (30.8)	6 (14)	0.165
**Azathioprine**	4 (30.8)	9 (20.9)	0.462
**Infliximab**	3 (23.1)	3 (7)	0.130
**Tocilizumab**	3 (23.1)	2 (4.7)	0.076
**Current and ex-smoker**	4 (33.3)	15 (38.5)	0.748
**Comorbidities, n (%)**	3 (23.1)	24 (55.8)	**0.038**

**n**, number of patients; **SD**, standard deviation; **CRP**, C-reactive protein**; ESR**, erythrocyte sedimentation rate; **DMARD**, disease-modifying antirheumatic drug.

Student’s t-test was used for the data conforming to normal distribution in the independent groups; the Mann–Whitney U test was used for the data that do not conform to normal distribution. Fisher’s exact test and chi-square test was used for categorical data, p < 0.05.

## Footnotes

1 Footnote: Republic of Turkey Ministry of Health General Directorate of Public Health (2021), COVID-19 (SARS-CoV-2 Infection) adult patient treatment guideline: Turkey [online]. Website: https://covid19.saglik.gov.tr/Eklenti/42169/0/covid19rehberieriskinhastayonetimivetedavi20122021v6pdf.pdf [accessed 12 Month 2021].

## References

[1] Harapan H Itoh N Yufika A Winardi W Keam S Coronavirus disease 2019 (COVID-19): A literature review Journal of Infection and Public Health 2020 13 5 667 673 10.1016/j.jiph.2020.03.019 32340833 PMC7142680

[2] Sanchez-Piedra C Diaz-Torne C Manero J Pego-Reigosa JM Rúa-Figueroa Í Clinical features and outcomes of COVID-19 in patients with rheumatic diseases treated with biological and synthetic targeted therapies Annals of the Rheumatic Disease 2020 79 7 988 990 10.1136/annrheumdis-2020-217948 PMC7307213 32503857

[3] Gianfrancesco M Hyrich KL Al-Adely S Carmona L Danila MI Characteristics associated with hospitalisation for COVID-19 in people with rheumatic disease: data from the COVID-19 Global Rheumatology Alliance physician-reported registry Annals of the Rheumatic Disease 2020 79 7 859 866 10.1136/annrheumdis-2020-217871 PMC7299648 32471903

[4] Keser G Aksu K Direskeneli H Takayasu arteritis: an update Turkısh Journal of Medical Sciences 2018 48 4 681 697 10.3906/sag-1804-136 30114347

[5] Emmi G Bettiol A Mattioli I Silvestri E Di Scala G SARS-CoV-2 infection among patients with systemic autoimmune diseases Autoimmunity Reviews 2020 19 7 102575 10.1016/j.autrev.2020.102575 32376395 PMC7200134

[6] Tomelleri A Sartorelli S Campochiaro C Baldissera EM Dagna L Impact of COVID-19 pandemic on patients with large-vessel vasculitis in Italy: a monocentric survey Annals of the Rheumatic Disease 2020 79 9 1252 1253 10.1136/annrheumdis-2020-217600 PMC7456557 32345617

[7] Arend WP Michel BA Bloch DA Hunder GG Calabrese LH The American College of Rheumatology 1990 criteria for the classification of Takayasu arteritis Arthritis & Rheumatology 1990 33 8 1129 1134 10.1002/art.1780330811 1975175

[8] Misra R Danda D Rajappa SM Ghosh A Gupta R Development and initial validation of the Indian Takayasu Clinical Activity Score (ITAS2010) Rheumatology (Oxford) 2013 52 10 1795 1801 10.1093/rheumatology/ket128 23594468

[9] Kerr GS Hallahan CW Giordano J Leavitt RY Fauci AS Takayasu arteritis Annals of Internal Medicine 1994 120 11 919 929 10.7326/0003-4819-120-11-199406010-00004 7909656

[10] Yek C Warner S Wiltz JL Sun J Adjei S Risk Factors for Severe COVID-19 Outcomes Among Persons Aged ≥18 Years Who Completed a Primary COVID-19 Vaccination Series - 465 Health Care Facilities, United States, December 2020 – October 2021 Morbidity and Mortality Weekly Report 2022 71 1 19 25 10.15585/mmwr.mm7101a4 34990440 PMC8735560

[11] Comarmond C Leclercq M Leroux G Marques C Le Joncour A Correspondence on ‘Impact of COVID-19 pandemic on patients with large-vessels vasculitis in Italy: a monocentric survey’ Annals of the Rheumatic Disease 2020 annrheumdis-2020-219407 10.1136/annrheumdis-2020-219407 33184046

[12] Banerjee S George M Young K Venkatachalam S Gordon J Effects of the COVID-19 Pandemic on Patients Living With Vasculitis ACR Open Rheumatology 2021 3 1 17 24 10.1002/acr2.11204 33784021 PMC7811691

[13] Favalli EG Monti S Ingegnoli F Balduzzi S Caporali R Incidence of COVID-19 in Patients With Rheumatic Diseases Treated With Targeted Immunosuppressive Drugs: What Can We Learn From Observational Data? Arthritis & Rheumatology 2020 72 10 1600 1606 10.1002/art.41388 32506699 PMC7300434

[14] Michelena X Borrell H López-Corbeto M López-Lasanta M Moreno E Incidence of COVID-19 in a cohort of adult and paediatric patients with rheumatic diseases treated with targeted biologic and synthetic disease-modifying anti-rheumatic drugs Seminars in Arthritis and Rheumatism 2020 50 4 564 570 10.1016/j.semarthrit.2020.05.001 32425260 PMC7229730

[15] Quartuccio L Valent F Pasut E Tascini C De Vita S Prevalence of COVID-19 among patients with chronic inflammatory rheumatic diseases treated with biologic agents or small molecules: A population-based study in the first two months of COVID-19 outbreak in Italy Joint Bone Spine 2020 87 5 439 443 10.1016/j.jbspin.2020.05.003 32445935 PMC7239017

[16] Russell CD Millar JE Baillie JK Clinical evidence does not support corticosteroid treatment for 2019-nCoV lung injury Lancet 2020 395 10223 473 475 10.1016/S0140-6736(20)30317-2 32043983 PMC7134694

[17] Lee N Allen Chan KC Hui DS Ng EK Wu A Effects of early corticosteroid treatment on plasma SARS-associated Coronavirus RNA concentrations in adult patients Journal of Clinical Virology 2004 31 4 304 309 10.1016/j.jcv.2004.07.006 15494274 PMC7108318

[18] Boekel L Kummer LY van Dam KPJ Hooijberg F van Kempen Z Adverse events after first COVID-19 vaccination in patients with autoimmune diseases Lancet Rheumatology 2021 3 8 e542 e545 10.1016/S2665-9913(21)00181-8 34179831 PMC8213359

